# Role of GBP1 in innate immunity and potential as a tuberculosis biomarker

**DOI:** 10.1038/s41598-022-15482-2

**Published:** 2022-06-30

**Authors:** Ting Shi, Linlin Huang, Yulin Zhou, Jianmei Tian

**Affiliations:** 1 Department of Infectious Diseases, Children’s Hospital of Soochow University, 303 Jingde Road, Suzhou, Jiangsu China; 2 Pediatric Intensive Care Unit, Children’s Hospital of Soochow University, Suzhou, China

**Keywords:** Biotechnology, Immunology, Diseases

## Abstract

Tuberculosis (TB) is a global health problem of major concern. Identification of immune biomarkers may facilitate the early diagnosis and targeted treatment of TB. We used public RNA-sequencing datasets of patients with TB and healthy controls to identify differentially expressed genes and their associated functional networks. *GBP1* expression was consistently significantly upregulated in TB, and 4492 differentially expressed genes were simultaneously associated with TB and high *GBP1* expression. Weighted gene correlation analysis identified 12 functional modules. Modules positively correlated with TB and high *GBP1* expression were associated with the innate immune response, neutrophil activation, neutrophil-mediated immunity, and NOD receptor signaling pathway. Eleven hub genes (*GBP1, HLA-B, ELF4, HLA-E, IFITM2, TNFRSF14, CD274, AIM2, CFB, RHOG*, and *HORMAD1*) were identified. The least absolute shrinkage and selection operator model based on hub genes accurately predicted the occurrence of TB (area under the receiver operating characteristic curve = 0.97). The GBP1-module-pathway network based on the STRING database showed that *GBP1* expression correlated with the expression of interferon-stimulated genes (*GBP5, BATF2, EPSTI1, RSAD2, IFI44L, IFIT3,* and *OAS3*). Our study suggests *GBP1* as an optimal diagnostic biomarker for TB, further indicating an association of the AIM2 inflammasome signaling pathway in TB pathology.

## Introduction

Tuberculosis (TB), caused by *Mycobacterium tuberculosis* (MTB) infection, remains one of the most significant health problems worldwide. The situation is dire, especially in the 30 high-TB-burden countries, which are predominantly low- and middle-income countries. According to the World Health Organization^1^, an estimated 10 million people were newly infected with TB in 2017. Of these, only 6.4 million were diagnosed and officially notified. It is estimated that 1.3 million people die from TB each year. In addition, more than one million children developed active TB in 2016, with 250,000 children dying from the disease^1^. Bacille Calmette-Guérin (BCG), a live attenuated vaccine, was first administered to humans in 1921 and has since been administered to more people than any other vaccine in history^2^. However, recent research has shown that BCG had only an estimated 19% effectiveness (95% confidence interval 8–29%) in protecting against MTB infection^3,4^, with almost no protective effect in adults. The mortality rate of TB in the pre-treatment era was nearly 50%, which was substantially higher than that in older children^5^. This number represents 10% of the total global burden of incident TB and 15% of the associated total mortality^1^. Therefore, there is an urgent need to identify new targets for prevention and treatment, which requires further research into the pathogenic mechanisms involved in MTB infection and the host response.

MTB can invade susceptible organisms via the respiratory tract, digestive tract, and skin damage. The bacterium is then swallowed by macrophages at the site of the infected tissue, and antigens are extracted into the lymphocytes. The innate immune system is the host’s first line of defense against invading pathogens, and the inflammasome plays an essential role in this process^6,7^. The guanylate-binding protein (GBP) family of interferon-inducible GTPases promotes antimicrobial immunity and consists of seven members in humans^8^. GBPs can target intracellular pathogens to mediate the defense response of the host via inflammasomes, oxidative responses, and autophagy^9–13^.

GBP1 is a 65-kD GTPase protein in the GBP family that plays a crucial role in innate immunity^14^, comprising a conserved region with an N-terminal globular GTPase domain and a C-terminal helical domain. Isoprenylation of the C-terminal CaaX box of GBP1 can achieve membrane anchoring^15^. Previous studies have shown that GBP1 promotes activation of inflammasomes to mediate pyroptosis and has dual membrane-disruptive actions to induce the atypical apoptosis of *Salmonella typhimurium* or *Toxoplasma gondii* in infected macrophages^16^. However, it is unclear whether GBP1 is associated with inflammasome activation in TB. Therefore, in this study, we searched for differentially expressed genes (DEGs) in TB patients based on public databases, and further investigated the DEGs associated with variations in *GBP1* expression and their functional networks. These findings can suggest *GBP1* as a new biomarker or therapeutic target, and provide further insight into the pathogenic mechanism of TB.

## Results

### Identification of DEGs in TB

**Figure 1:**
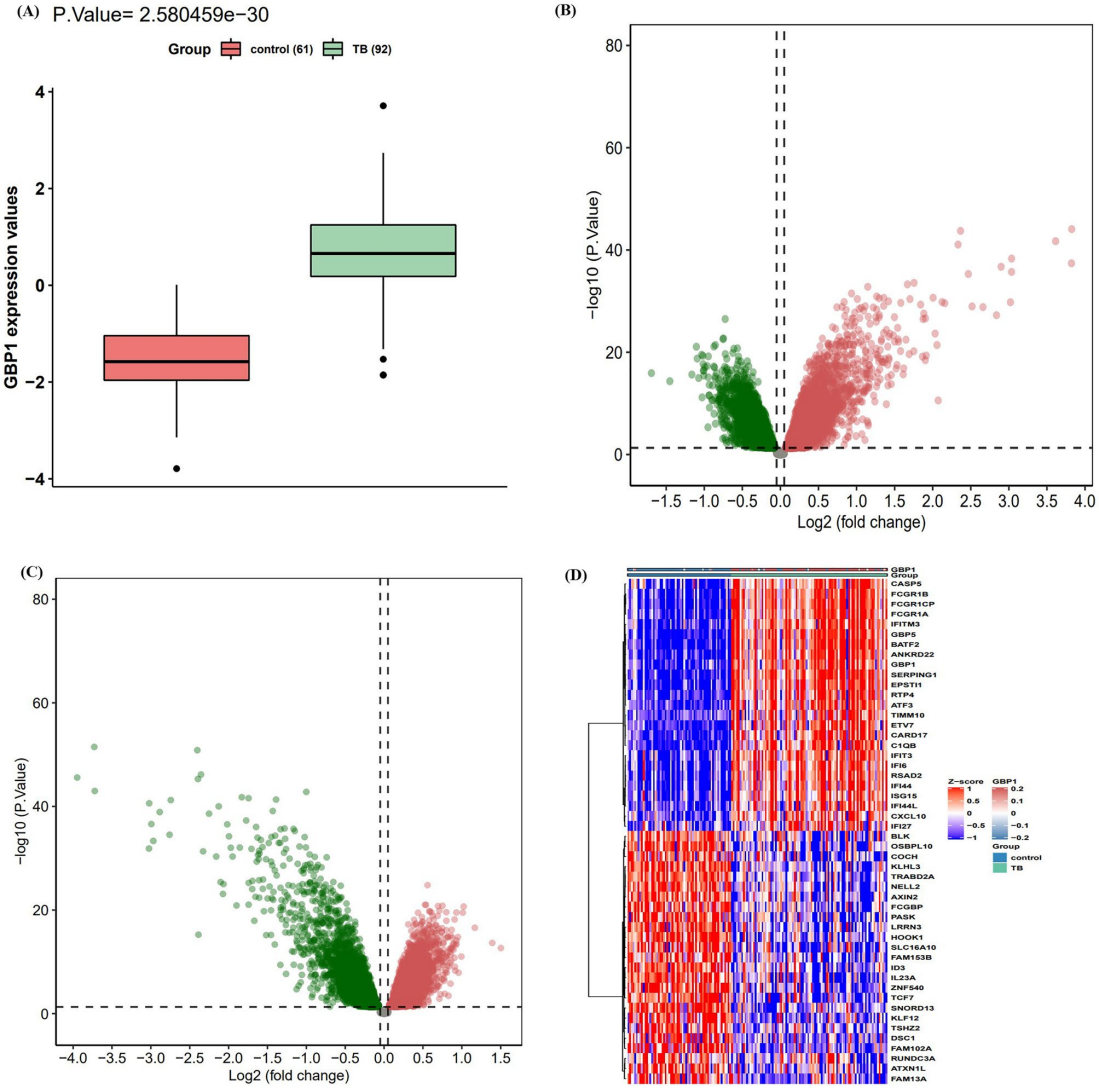
Differential expression gene analysis. (**A**) GBP1 is up-regulated in TB (P = 2.580e-30, 92 TB patients and 61 normal people). (**B**) Volcano plot of the TB-control, red represents up-regulated genes, blue represents down-regulated genes, and black represents no significantly differentially expressed genes. (**C**) Volcano plot of the GBP1-low/high, red represents up-regulated genes, blue represents down-regulated genes, and black represents no significantly differentially expressed genes. (**D**) A heatmap of 25 most up-regulated and 25 most down-regulated genes.

Data were extracted from the Gene Expression Omnibus (GEO) database, including the gene expression matrix, clinical characteristics, and probe set (https://www.ncbi.nlm.nih.gov/geo/). In the GSE83456 dataset (including 92 TB patients and 61 healthy controls), we found that *GBP1* expression was significantly upregulated in TB samples compared to the control samples [P = 2.580e-30 and log fold change (FC) = 2.158] (Fig. 1A). This suggested that high expression of *GBP1* may be associated with TB. Along with *GBP1*, compared to the control samples, we identified a total of 5302 DEGs in TB, 3274 of which were upregulated and 2028 of which were downregulated (Fig. 1B). Moreover, there were 5023 DEGs in the *GBP1*-high samples compared to the *GBP1*-low samples (divided according to the median GBP1 expression level), including 2129 upregulated genes and 2894 downregulated genes (Fig. 1C). A total of 4492 genes were found at the intersection of the two DEG sets, which indicated a relation to the expression of *GBP1* and to TB. The 25 most strongly upregulated and 25 most strongly downregulated genes in TB are visualized using a heat map in Fig. 1D. The upregulated genes mainly included interferon-stimulated genes (ISGs) and inflammasome activation-related genes (*CASP5* and *CARD17*).

### Module associated with TB

**Figure 2:**
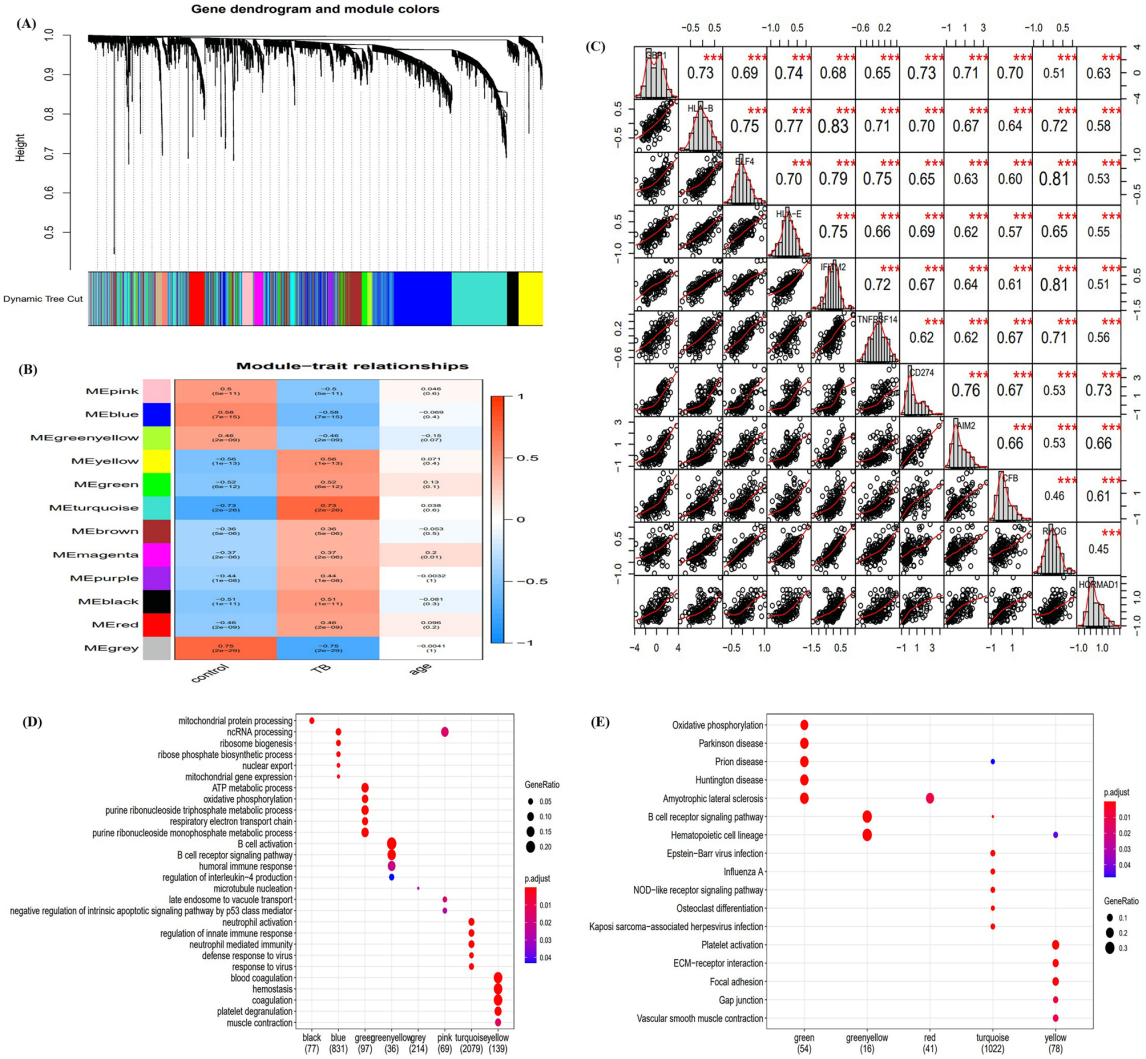
Weighted correlation network analysis. (**A**) Recognition module, each module was given an individual color as identifiers, including 12 different modules. (**B**) Correlation heat map of gene modules and phenotypes, the red was positively correlated with the phenotype, blue was negatively correlated with the phenotype. (**C**) The correlation between GBP1 and hub genes,red indicated negative correlation and blue indicated positive correlation. (**D**) Biological processes of module genes, the significance of enrichment gradually increased from blue to red, and the size of the dots indicates the number of differential genes contained in the corresponding pathway^17–19^. (**E**) KEGG pathways analysis of module genes. The significance of enrichment gradually increased from blue to red, and the size of the dots indicated the number of differential genes contained in the corresponding pathway^17–19^.

Weight gene correlation network analysis **(**WGCNA) was performed using the expression profile of genes commonly associated with TB and *GBP1* expression to identify the key module with the strongest correlation to TB. Among the 12 modules identified (Fig. 2A), the turquoise module was significantly positively correlated with TB (correlation coefficient = 0.73, P = 2e-26; Fig. 2B). According to the criteria of a gene significance (GS) value > 0.6 and module membership (MM) > 0.9, 11 genes were identified as hub genes (*GBP1, HLA-B, ELF4, HLA-E, IFITM2, TNFRSF14, CD274, AIM2, CFB, RHOG*, and *HORMAD1*). Correlation analysis showed that *GBP1* was strongly correlated with the other hub genes, and the correlation coefficients between *GBP1* and *HLA-B, HLA-E, CD274, AIM2*, and *CFB* were all > 0.7 (Fig. 2C). Module function enrichment analysis further showed that turquoise module genesare significantly involved in biological processes related to regulation of the innate immune response, neutrophil activation, and neutrophil-mediated immunity (Fig. 2D), and the module was significantly enriched in the NOD receptor signaling pathway (Fig. 2E).

### Network of *GBP1*

**Figure 3:**
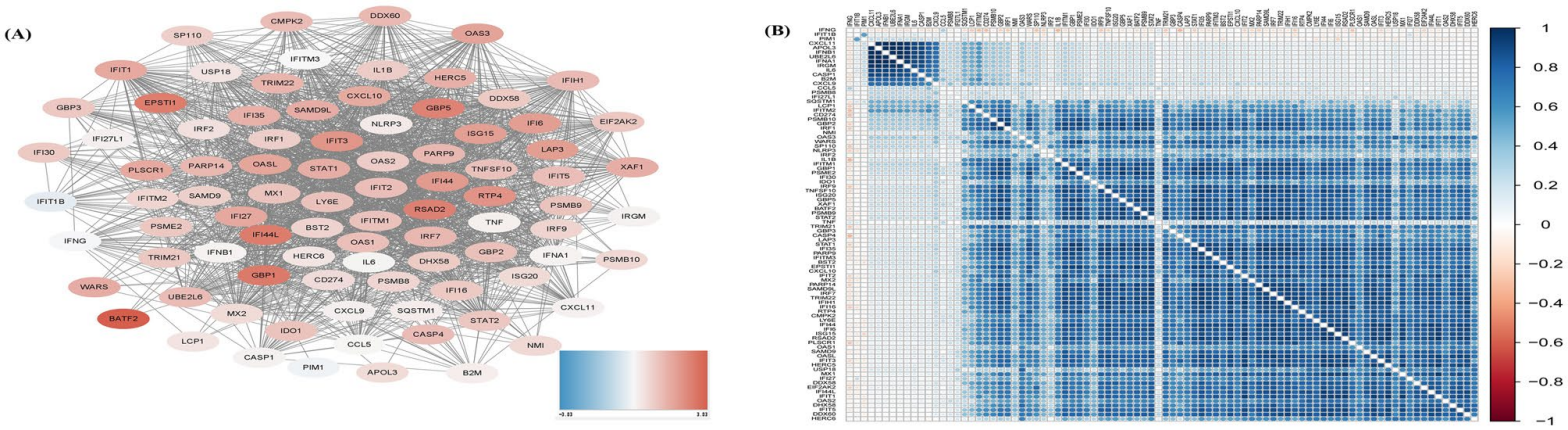
Network construction of GBP1 modular genes. (**A**) The PPI network construction of GBP1 and each modular genes based on the STRING database, red was up-regulated genes and blue was down -regulated genes, the value was adjusted by the false discovery rate (FDR). (**B**). The correlation between GBP1 and modular genes, red indicates negative correlation and blue indicates positive correlation.

We extracted the genes found to interact with *GBP1* in the modules based on the STRING database that also showed differential expression (logFC) in TB (Fig. 3A). The correlation analysis of *GBP1* and these genes in the GSE83456 dataset was consistent with the results of the network construction diagram (Fig. 3B). According to Kyoto Encyclopedia of Genes and Genomes (KEGG) pathway enrichment analysis, these significant genes were enriched in the RIG-I-like receptor signaling pathway. Both network and correlation analyses showed that *GBP1* is strongly correlated with the interferon-stimulated genes *BATF2, GBP5, EPSTI1*, *RSAD2, IFI44L, IFIT3*, and *OAS3*.

### Combined predictive marker of TB

**Figure 4:**
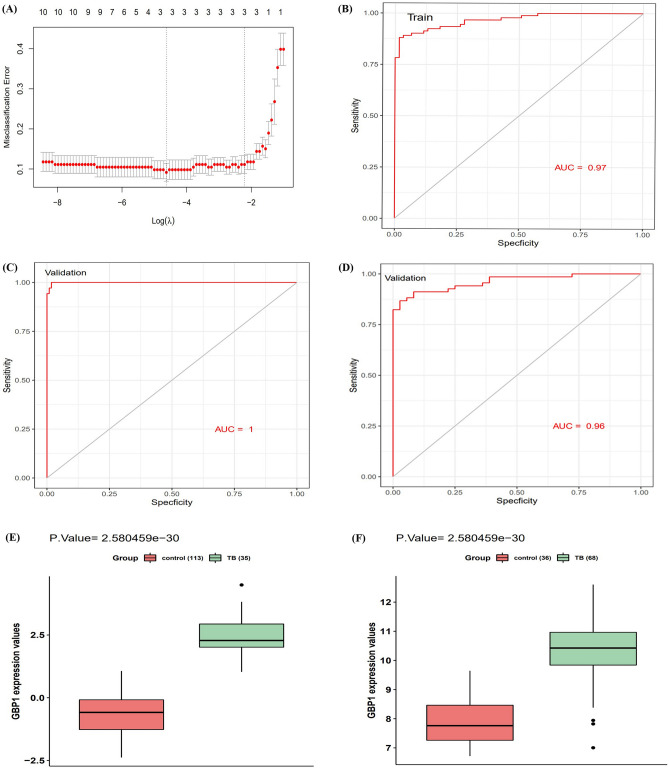
A model for predicting TB and verification of expression of GBP1 in differential datasets. (**A**) LASSO model. (**B**) ROC curves analysis of train set (GSE83456). (**C**) ROC curves analysis of validation (GSE42834). (**D**) ROC curves analysis of validation (GSE19491). (**E**) GBP1 is up-regulated in TB (P = 2.580e-30, 35 TB patients and 113 normal samples are contained). (**F**) GBP1 is up-regulated in TB (*P* = 2.580e-30, 68 TB patients and 36 normal samples are contained).

We extracted the expression profiles of the hub genes to construct a least absolute shrinkage and selection operator (LASSO) model (Fig. 4A). Using the LASSO method, four genes were identified with non-zero regression coefficients and a minimum lambda value of 0.09878891. The model index of these four genes was defined as *GBP1* × 0.1568 + *HLA − B* × 0.0845 + *ELF4* × 0.0965 + *TNFRSF14* × 0.0279. The area under the receiver operating characteristic (ROC) curve (AUC) (Fig. 4B) of this four gene-based model was 0.97 in the training set, indicating good predictive ability; thus, the LASSO model may be used as a biomarker of TB. This was further validated in two independent GEO datasets used as validation set (GSE42834 and GSE19491) with AUC values of 0.998 and 0.96, respectively (Fig. 4C and D). Furthermore, we found that *GBP1* was also highly upregulated in the blood samples of TB patients in the GSE42834 and GSE19491 datasets (Fig. 4E, F). This indicated that *GBP1* and its related hub genes are strongly associated with TB and could potentially serve as diagnostic biomarkers.

## Discussion

According to the 2018 Global Tuberculosis Report of the World Health Organization^1^, there were approximately 10 million new cases of TB (5.8 million men, 3.2 million women, and 1 million children) worldwide in 2017, leading to approximately 1.6 million deaths. At present, the global control of TB faces major challenges, including the lack of effective vaccines, rapid and sensitive diagnostic methods, and effective treatment strategies for drug-resistant bacteria. Studying the body’s immune regulation mechanism against MTB will help to identify new methods for the diagnosis and treatment of TB. Previous studies have focused on the mechanism of adaptive immunity in TB, specifically in T helper cells. In recent years, increasing attention has been paid to the role of the innate immune system. Innate immunity is not only the first barrier of the body’s immune system but is also an indispensable factor in initiating adaptive immunity. Innate immune components can quickly recognize foreign antigens depending on the expression of various pattern recognition receptors^20^.

In the present study, we found that *GBP1* expression was generally upregulated in TB patients, suggesting that high expression of *GBP1* may play a role in fighting MTB infection. Furthermore, ISGs and inflammasome-activation genes such as *CASP5* and *CARD17* were highly expressed in both the TB and GBP1-high groups, suggesting that *GBP1* is associated with the interferon signaling pathway and inflammasome activation in TB. According to WGCNA, a total of 12 modules associated with TB were identified. Moreover, according to the criteria of GS > 0.6 and MM > 0.9, 11 genes were identified as hub genes in the TB module, including *GBP1, HLA-B, ELF4, HLA-E, IFITM2, TNFRSF14, CD274, AIM2, CFB, RHOG*, and *HORMAD1*. Among them, *HLA-B* and *HLA-E* belong to the major histocompatibility complex class I protein complex and are involved in the presentation of foreign antigens to CD8( +) T cells in TB^21,22^. *ELF4* plays a role in the development and function of natural killer (NK) and NK T cells, as well as in innate immunity, and controls the proliferation and homing of CD8 + T cells via the Kruppel-like factors KLF4 and KLF2^23^; however, its mechanism of action in TB has not yet been explored. *IFITM* encodes a transmembrane protein induced by interferon that can restrict the intracellular growth of MTB^24^. MTB can promote regulatory T-cell expansion via the induction of programmed death-1 ligand 1 (CD274) on dendritic cells^25^. *AIM2* encodes a type of inflammasome that can recognize intracellular double-stranded DNA (dsDNA) and mediate the release of the pro-inflammatory cytokines interleukin (IL)-1B and IL-18^26^. Furthermore, we found that *GBP1* expression was highly correlated with the expression levels of *HLA-B, HLA-E, CD274*, *AIM2*, and *CFB*, which further supported that *GBP1* may play an important role in innate immunity such as antigen presentation and AIM2 inflammasome activation.

We further explored the TB and *GBP1*-related biological processes and pathways. Functional enrichment analysis showed that modules with a strong correlation to TB were significantly involved in several immune-related biological processes, including the innate immune response, neutrophil activation, neutrophil-mediated immunity, and the NOD receptor (NLR) signaling pathway. Thus, interactions between GBP1 and AIM inflammasome-related NLRs could contribute to TB infection and pathogenesis.

**Figure 5:**
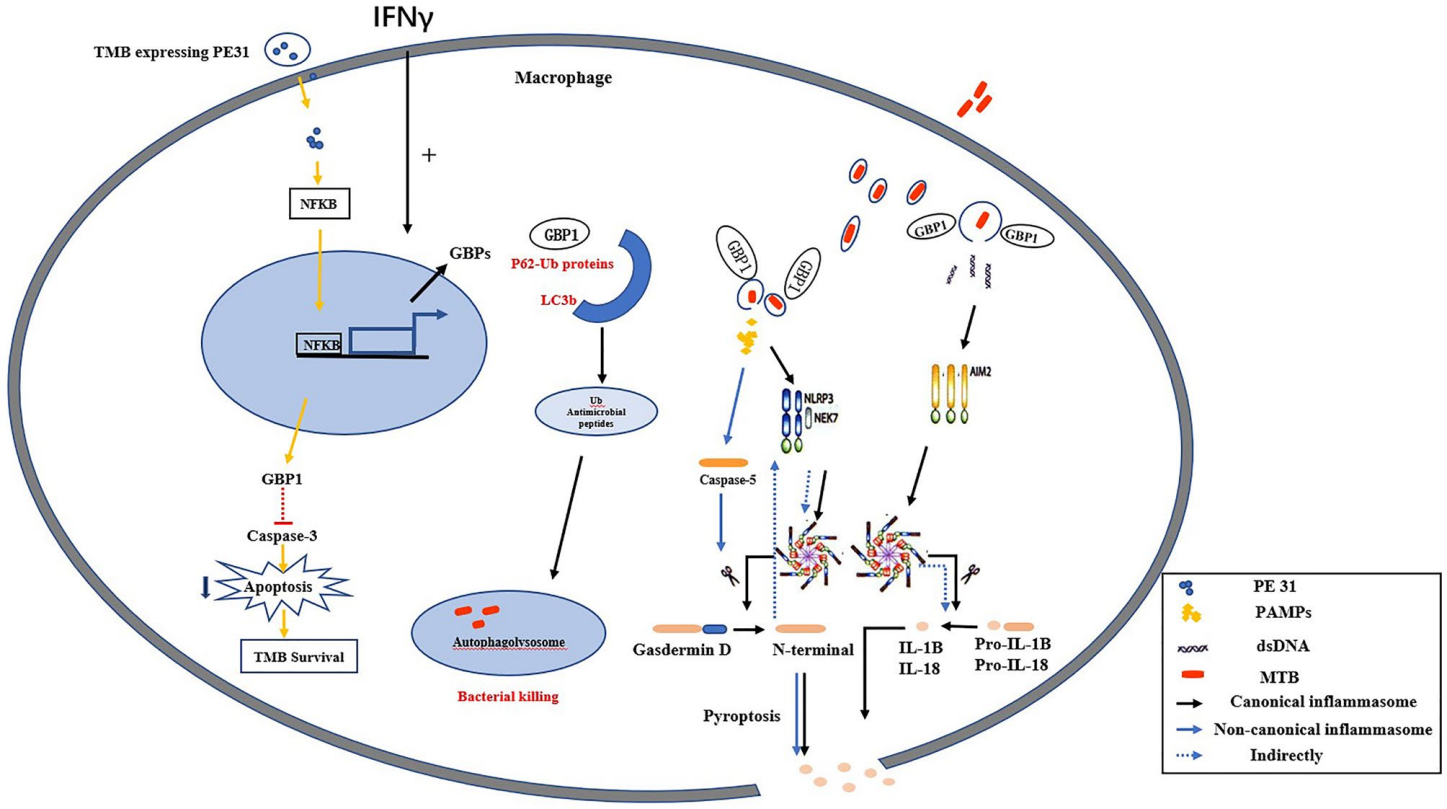
Potential mechanism of high expression of GBP1 associated with TB in macrophage.

Multiple studies have demonstrated that GBP1 plays an important role in MTB infection; however, its mechanism of action remains controversial. Interferon-gamma was found to initiate transcription of the mouse *Gbps* gene, which is mobilized as part of a new inducible antimicrobial program to combat infection^27^. Moreover, GBP7 participates along with GBP1 in the trafficking of mono-ubiquitinated protein cargo to autolysosomes to generate ubiquitin (Ub)-derived antimicrobial peptides in TB^27^. Specifically, GBP1 binds to p62-Ub for delivery to LC3b + membranes and for engulfment, and is then recycled after p62-Ub delivery. GBP7 recruits ATG4B for LC3b + membrane elongation and closure around this cargo to generate lytic peptides that kill mycobacteria after fusion with phagosomes^27^. Zak et al.^28^ also showed that GBP1 plays a protective role during TB infection. Moreover, Ali et al.^29^ suggested that Ms_PE31 of MTB activated the NF-κB signaling pathway to mediate the expression of IL-10 and GBP1 protein in macrophages. They further suggested that IL-10 and GBP1 might inhibit the activation of caspase-3 and lead to the attenuation of macrophage apoptosis, thereby fostering the intracellular survival of mycobacteria and establishment of infection (Fig. 5). In summary, the role of GBP-1 in MTB infection remains unclear.

MTB can infect cells such as macrophages and dendritic cells, and subvert them to propagate and disseminate within the host. MTB releases components such as bacterial DNA into the cytosol via mechanisms that are still not completely understood^30^. AIM2 recognizes intracellular dsDNA and mediates the release of the pro-inflammatory cytokines IL-1B and IL-18, which has been associated with protection against pulmonary tuberculosis (PTB)^26^. However, no study has yet explored whether GBP1 is involved in activation of the AIM2 inflammasome in TB. The dual membrane-disruptive actions of GBP1 in *Salmonella* Typhimurium or *T. gondii* infection promotes the detection of dsDNA by AIM2 and induces cell pyroptosis^16,31^. Pyroptosis has also been found to be significantly associated with TB and to play a crucial role in the host defense against intracellular MTB^30,32–34^. Our study provides support for these previous findings, further revealing the interaction between GBP1 and the AIM2 inflammasome signaling pathway.

In addition, the expression profiles of hub genes were extracted to construct a LASSO model, in which the regression coefficients of the four genes were non-zero values. ROC curve analysis showed that the LASSO model had a high AUC value, showing strong discrimination against TB in healthy individuals, indicating that it may serve as a biomarker of TB. This was verified using an independent dataset. We further found that *GBP1* expression was consistently upregulated in various datasets, which supports the involvement of high *GBP1* expression in the pathogenesis of TB.

In summary, this study demonstrates that bioinformatics analysis can reveal important molecular pathways in TB. However, the potential key pathways and genes require further verification in molecular experiments.

## Methods

### Data

**Figure 6:**
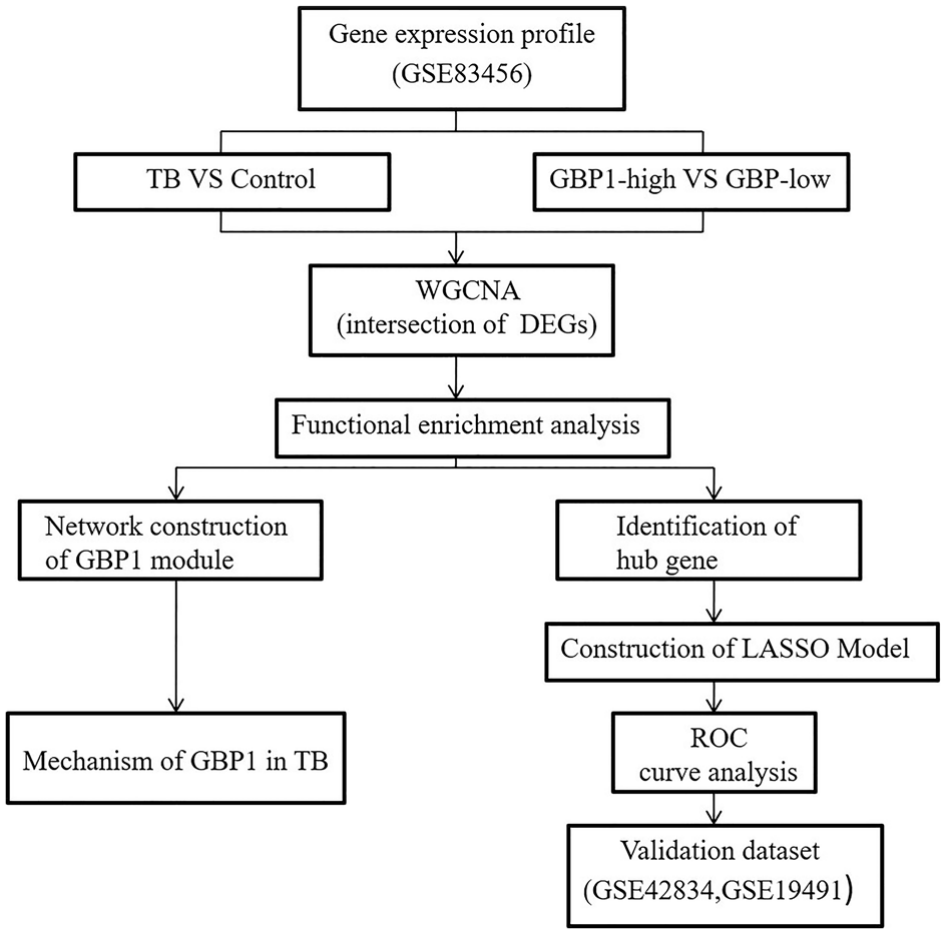
The workflow of the study.

The R/Bioconductor package GEOquery was used to extract the GSE83456, GSE42834, and GSE19491 data from the GEO database. The GSE83456 dataset includes whole blood samples from 92 TB patients (47 with extrapulmonary tuberculosis and 45 with PTB) and 61 healthy individuals, which was used to explore the potential involvement of *GBP1* in TB. There was no difference in sex or age distribution between the TB and control groups (Table 1). In addition, the GSE42834 and GSE19491 datasets were used for validation, including samples from 35 PTB patients and 113 controls and 68 PTB patients and 36 controls, respectively. The workflow of the study is illustrated in Fig. 6. The original studies for compiling these datasets were approved by the Central London 3 Research Ethics Committee (09/H0716/41); CPP Sud-Est IV, France, CCPPRB; and les Hôpitaux Universitaires Pitié Salpêtrière, Paris. All participants provided written informed consent.

**Table 1:** The general characteristics of participants in the three datasets.

Datasets	TB group	Control group	*P*
**GSE83456**			
N	92	61	
Gender (female: male)	37:55	28:33	0.486
Age (years)	37.5 ± 13.4	35.8 ± 11.6	0.405
**GSE42834**			
N	35	113	
Gender (female: male)	16:19	72:41	0.058
**GSE19491**			
N	68	36	
Gender (female: male)	26:42	22:14	0.026
Age (years)	36.7 ± 14.7	30.49 ± 7.9	0.008

Abbreviations: TB, tuberculosis; N, number.

### Gene expression profiling of datasets

The samples were the ones used to generate the described GEO datasets. Three milliliters of whole blood were collected from the children by standard phlebotomy before treatment and vigorously mixed immediately. RNA was isolated from the whole blood using a MagMAX-96 Blood RNA Isolation kit (Applied Biosystems/Ambion). Total RNA was globin-reduced using the GLOBINclear 96-well format kit (Applied Biosystems/Ambion). An Agilent 2100 Bioanalyzer (Agilent, Palo Alto, CA, USA) was used to assess the total and globin-reduced RNA integrity. RNA targets (cRNA) were amplified using an Illumina CustomPrep RNA amplification kit (Ambion, Austin, TX, USA). Labeled cRNA was hybridized to Sentrix HT12 V3 BeadChip arrays overnight, followed by washing, blocking, staining, and scanning. Signal intensity analysis (per-chip normalization) was performed using Illumina BeadStudio version 2 software^35^. All gene expression measurements were log_2_-transformed and normalized according to the manufacturer’s instructions.

### Identification of DEGs between TB and control groups

The limma package in R was used for differential expression analysis. Using the median expression level of *GBP1* as the cut-off point, the subjects were divided into *GBP1*-high and *GBP1*-low expression groups. We used the lmFit and eBayes functions in the limma package to identify DEGs between the TB and control groups and between the *GBP1*-high and *GBP1*-low groups. *P* < 0.05 adjusted by the false discovery rate was considered to indicate a significant difference in expression level.

### WGCNA

WGCNA was performed for genes that were commonly upregulated or downregulated in both the TB vs. control and *GBP1*-low vs. *GBP1*-high comparisons. We identified potential functional modules for biological function characterization of each subgroup. Subsequently, the soft thresholding power value (power = 7) was screened during module construction using the soft threshold function. The distance between each gene pair was evaluated on the basis of the similarity of the topological overlap matrix. In addition, average and dynamic methods were used for hierarchical clustering analysis, and then the clustering tree and gene modules were constructed. After merging the original 12 modules based on their similarities, Spearman correlation analysis in the WGCNA package was used to calculate the correlation coefficients and associated p-values between clinical features and functional modules. The clusterProfiler package in R was used to perform functional enrichment analyses of these functional modules.

### Identification of hub genes and construction of the GBP1-module-pathway network

In WGCNA, GS is defined as the correlation between a gene and a phenotype and MM is defined as the measure of importance of a gene in a module according to the formula MM(i) = cor(x i, ME). In this study, a gene with GS > 0.6 and MM > 0.9 was defined as a hub gene among the candidate gene modules. In addition, DEGs that interact with *GBP1* were extracted from the STRING database (https://STRING-db.org/). The correlation between the DEGs interacting with *GBP1* and hub genes was then explored.

### LASSO model and ROC curve analysis

LASSO has strong predictive value and is considered suitable for selecting the best features for high-dimensional data. We extracted the expression profiles of hub genes to construct the LASSO model for distinguishing TB and healthy samples using the glmnet package. Regression coefficients from LASSO analysis were used to create a model index for each sample to weigh the expression values of selected genes using the following formula: index = ExpGene1*Coef1 + ExpGene2*Coef2 + ExpGene3*Coef3 + …., where “Coef” is the regression coefficient of a gene, derived from the LASSO Cox regression, and “Exp” represents the expression values of the gene. To evaluate the ability of the LASSO model to identify TB, we used the pROC package to perform ROC curve analysis on the test (GSE83456) and validation (GSE42834 and GSE19491) datasets.

All methods were performed in accordance with the relevant guidelines and regulations.

## Data Availability

The data supporting the findings of this study are available from the corresponding authors upon reasonable request (2231365607@qq.com; zhouyulin@163.com).
